# Effects of 2 months of methylphenidate on energy expenditure in individuals with obesity: A randomized, double‐blind, placebo‐controlled pilot study

**DOI:** 10.14814/phy2.16085

**Published:** 2024-06-26

**Authors:** Kurt McInnis, Éric Doucet, Kaamel Hafizi, Fatmé El Amine, Brandon Heidinger, Jameason D. Cameron, Shakibasadat BaniFatemi, Philippe Robaey, Régis Vaillancourt, Gary S. Goldfield

**Affiliations:** ^1^ School of Human Kinetics University of Ottawa Ottawa Ontario Canada; ^2^ Children's Hospital of Eastern Ontario Research Institute Ottawa Ontario Canada; ^3^ Department of Pharmacy, Children's Hospital of Eastern Ontario Ottawa Ontario Canada; ^4^ BCE Pharma Saint‐Georges Quebec Canada

**Keywords:** adaptive thermogenesis, energy balance, energy expenditure, methylphenidate, obesity, weight loss

## Abstract

Methylphenidate (MPH) has been previously shown to increase resting energy expenditure (REE) in individuals of normal weight; however, the effects on individuals living with obesity are currently unknown. Ten individuals living with obesity were randomly assigned to undergo 60 days of MPH administration with a daily dose of 0.5 mg/kg body weight or a placebo control. REE was measured before and after the 60‐day intervention. There was a trend toward significance for group × time interaction on REE (*p* = 0.082) with a large effect size (*η* ^2^ = 0.331), with MPH administration increasing REE compared to a decrease in placebo control. Preliminary findings from this pilot study show that MPH has the potential to counter the adaptive thermogenic process commonly seen in weight loss. This is a unique finding among pharmacotherapies, as no approved obesity drugs measurably impact REE.

## 1 INTRODUCTION

Weight loss requires sustained negative energy balance, with pharmacotherapy being more effective than diet and exercise in promoting negative energy balance and ensuing weight loss (Khera et al., 2016). As weight loss progresses, humans display an increase in appetite (Doucet et al., 2000) and decrease in energy expenditure (EE) which is often precipitated downward at a greater rate than expected (Doucet et al., 2001). Therefore, identifying therapeutic avenues with the potential to correct these bio‐behavioral adaptations to weight loss on energy balance are appealing. We have previously reported that Methylphenidate (MPH) reduces energy intake (EI) and suppresses appetite acutely in individuals with normal weight (Goldfield et al., 2007), as well as over a 2‐months intervention in individuals living with obesity (El Amine et al., 2022). What is more, we have also shown that fast‐release MPH acutely increases resting EE (REE) by approximately 7% and the thermic effect of feeding (TEF) by ~5% in individuals with normal weight (Lorello et al., 2008). MPH is unique in this aspect, as no other approved obesity drugs to our knowledge have measurably impacted EE (Khera et al., 2016). To our knowledge, there are no controlled studies on the impact of MPH on physical activity EE (PAEE) in individuals without attention‐deficit/hyperactivity disorder. The relatively low incidence of intolerability (Stuhec et al., 2019), ease of administration through oral ingestion, lower cost, and indication for pediatric populations makes MPH a compelling compound to study for its potential to counteract adaptive thermogenic responses and lead to weight loss. To date, whether the stimulating effects of MPH on REE are also present in individuals living with obesity, and whether these increases can be sustained with chronic ingestion, remains to be determined. The objective of this pilot study was to determine whether orally ingested MPH can produce sustained elevation of REE over the course of a 60‐day intervention in individuals living with obesity.

## 2 METHODS

Participants included in this study were a subsample from a previously published study (El Amine et al., 2022). Ten individuals (*n* = 5 men) living with obesity were randomly assigned to undergo a 60‐day randomized, double‐blind, placebo‐controlled, parallel‐arm clinical trial pilot study using either 0.5 mg/kg body weight of MPH or a placebo control (lactose monohydrate) (ClinicalTrials.gov Identifier: NCT02754258). The study recruitment and conduct were per the Consolidated Standards of Reporting Trials (CONSORT) guidelines (Schulz et al., 2010). In this experiment, the drug was administered twice per day, and titrated over 7 days in gradual 15% daily increments, starting at 0.25 mg/kg BW until reaching the best tolerated dose (up to 0.5 mg/kg BW). All participants tolerated the maximal dose, therefore after the 7‐day titration period all participants in the MPH group ingested a daily dose of 0.5 mg/kg of BW taken twice daily as previously described (2). REE was measured after a 12‐h overnight fast, at baseline before MPH administration (Day 0) and the intervention's conclusion (Day 60). TEF was measured for 30 min of each hour for a 4 h period after the consumption of a standardized meal. REE and TEF were measured using a Vmax Encore 29N metabolic cart (Sensor Medics Corporation) and dual‐energy x‐ray absorptiometry scans (Lunar Prodigy; General Electric) assessed body composition as previously described (Hintze et al., 2018). PAEE was measured using an Actical accelerometer (Actical‐Mini, Mitter Co. Inc.), worn for 7 days at baseline and the week preceding 60‐day post‐intervention. The study received approval from the Research Ethics Boards at the University of Ottawa and Children's Hospital of Eastern Ontario. Written informed consent was obtained from all participants and the study conduct adhered to the guidelines in the Declaration of Helsinki.

## 3 STATISTICAL APPROACH

REE, TEF, and PAEE data were analyzed using repeated measures analysis of variance (ANOVA) with group (MPH vs. placebo) as the between‐subjects independent variable, and time (baseline vs. 60 days post) as the within‐subjects independent variable, and group × time interaction indicative of a treatment effect, with alpha at *p* < 0.05. Effect size is also reported as eta squared, *η* ^2^, whereby values above 0.01, 0.06, and 0.14 were considered small, medium, and large effects, respectively. Data are presented as mean ± standard deviation (SD) unless otherwise specified. Statistical analyses were performed using Statistical Product and Service Solutions software, version 29.0 (Chicago, IL).

## 4 RESULTS

There were no significant differences in age, sex, BMI, body weight, or composition between the MPH and placebo groups at baseline (Table 1 **)**.

**TABLE 1:** Participant characteristics at baseline.

Variable	Placebo (*n* = 5) mean (SD)	MPH (*n* = 5) mean (SD)	*p*
Age (years)	29.0 (7.9)	26.6 (8.5)	0.656
Sex (M/F)	(2/3)	(3/2)	0.580
Height (cm)	171.3 (9.7)	168.7 (11.6)	0.711
Body weight (kg)	112.3 (20.1)	102.4 (25.5)	0.515
Fat mass (kg)	50.2 (14.1)	45.5 (10.2)	0.564
Fat‐free mass (kg)	57.3 (11.1)	53.4 (15.9)	0.366
BMI (kg/m^2^)	38.0 (2.8)	35.6 (5.7)	0.432
REE (kcal/day)	2122 (270)	1866 (308)	0.246

Abbreviations: BMI, body mass index; MPH, methylphenidate; REE, resting energy expenditure.

**FIGURE 1:**
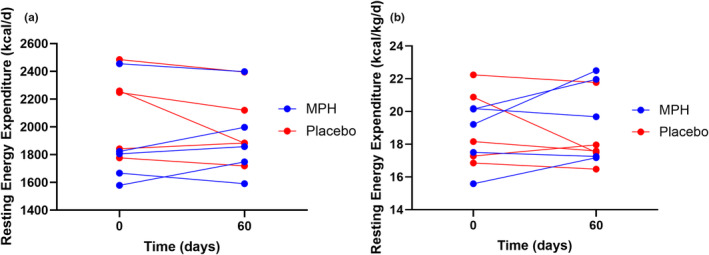
(a) Change in absolute resting energy expenditure values after a 60‐day intervention of MPH or placebo administration relative to their baseline values. MPH, methylphenidate. (b) Change in resting energy expenditure values relative to body weight after a 60‐day intervention of MPH or placebo administration relative to their baseline values. MPH, methylphenidate.

There was a trend toward significance for the group × time interaction on REE (*p* = 0.082), whereby the absolute REE increased by an average of 52.3 ± 106.5 kcals following MPH administration, compared to a decrease of 122 ± 139.0 kcal for placebo, with a large effect size (*η* ^2^ = 0.331). Moreover, relative REE (REE/body weight) increased by 6.5% from baseline to Day 60 for the MPH group compared to a 4% decrease in the placebo group (*p* = 0.074), again reflecting a large effect size (*η* ^2^ = 0.346) (Figure 1). Finally, REE relative to fat‐free mass increased with MPH administration (35.9 ± 5.2–37.6 ± 5.5 kcal/kg/day) and decreased with placebo (37.9 ± 7.5–36.3 ± 5.1 kcal/kg/day) (*p* = 0.150), reflecting a large effect size (*η* ^2^ = 0.241). No group differences existed for TEF or physical activity EE (data not shown).

Based on the preliminary data provided by this study, the correlations of repeated measures (*p* = 0.700 for placebo and *p* = 0.900 for MPH), α error probability of 0.05 and power of 0.80, a sample size of 9 individuals per group is recommended to achieve adequate statistical power.

## 5 DISCUSSION

MPH has been shown to decrease EI (Goldfield et al., 2007) in acute doses and decrease appetite with both acute administrations among individuals with normal weight (Goldfield et al., 2007) and chronic administrations among those living with obesity (El Amine et al., 2022). Based on these pilot data and the large effect sizes shown, we highlight the possibility for the first time that MPH may chronically increase REE in participants with obesity, an effect that was apparent even in the presence of a 2.7 kg weight loss, countering the ubiquitous adaptive thermogenic process that is typically accompanied by reductions in REE both acutely and chronically (Hintze et al., 2018). This correction of bio‐behavioral adaptations to weight loss is unique among pharmacotherapies, as no approved drugs have demonstrated such an effect on REE, indicating MPH has the potential to tilt the scales of energy balance by decreasing EI and increasing REE, which is ideal to potentiate weight loss. The 2.7 kg average weight loss previously described (El Amine et al., 2022) was achieved in the absence of diet or exercise, a result comparable to other pharmacotherapy interventions at 8 weeks (Khera et al., 2016). Importantly, the tolerability of MPH within both our cohort, as well as in other assessments, is comparable or better than other available pharmacotherapy interventions (El Amine et al., 2022; Khera et al., 2016; Stuhec et al., 2019). The increase in REE observed also occurred without a compensatory decrease in PAEE, a common intended effect with individuals with attention deficit/hyperactivity disorder (Butte et al., 1999).

These preliminary findings, although novel and compelling due to their large effect sizes, warrant further study with larger and more adequately powered sample sizes to clearly identify the effects of chronic administration of MPH on REE and the role this plays in weight loss and maintenance of weight loss among individuals living with obesity.

## AUTHOR CONTRIBUTIONS

Conceptualization (ED, GSG, PR, JDC, RV), methodology (ED, GSG, PR, JDC, RV), formal analysis (ED, GSG, KM), investigation (PR, JDC, FE, KH, SB), resources (JDC, FE, KH, SB), data curation (ED, GSG, JDC, FE, KM), writing‐original (KM, ED, GSG), writing‐review (all author), supervision (ED, GSG), administration (ED, GSG, JDC), and funding (ED, GSG, RV).

## CONFLICT OF INTEREST STATEMENT

The authors declare there are no competing interests.

## ETHICS STATEMENT

This study received approval from the Research Ethics Boards at the University of Ottawa and Children's Hospital of Eastern Ontario. Written informed consent was obtained from all participants and the study conduct adhered to the guidelines in the Declaration of Helsinki.

## Data Availability

The data that support the findings of this study are available on request from the corresponding author. The data are not publicly available due to privacy or ethical restrictions.
